# Neuronal CXCL10/CXCR3 Axis Mediates the Induction of Cerebral Hyperexcitability by Peripheral Viral Challenge

**DOI:** 10.3389/fnins.2020.00220

**Published:** 2020-03-24

**Authors:** Tiffany J. Petrisko, Jenna Bloemer, Priyanka D. Pinky, Sriraja Srinivas, Ryan T. Heslin, Yifeng Du, Sharay E. Setti, Hao Hong, Vishnu Suppiramaniam, Gregory W. Konat, Miranda N. Reed

**Affiliations:** ^1^Departments of Biochemistry and Neuroscience, West Virginia University School of Medicine, Morgantown, WV, United States; ^2^Drug Discovery and Development, School of Pharmacy, Auburn University, Auburn, AL, United States; ^3^Department of Pharmacy, The First Affiliated Hospital of Xiamen University, Xiamen, China; ^4^Key Laboratory of Neuropsychiatric Diseases, Jiangsu Key Laboratory of Drug Discovery for Metabolic Diseases, and State Key Laboratory of Natural Medicines, China Pharmaceutical University, Nanjing, China; ^5^Center for Neuroscience Initiative, Auburn University, Auburn, AL, United States

**Keywords:** acute phase response, hyperexcitability, polyinosinic-polycytidylic acid, CXCL10, CXCR3, synaptic transmission, synaptic plasticity

## Abstract

Peripheral infections can potently exacerbate neuropathological conditions, though the underlying mechanisms are poorly understood. We have previously demonstrated that intraperitoneal (i.p.) injection of a viral mimetic, polyinosinic-polycytidylic acid (PIC) induces a robust generation of CXCL10 chemokine in the hippocampus. The hippocampus also features hyperexcitability of neuronal circuits following PIC challenge. The present study was undertaken to determine the role of CXCL10 in mediating the development of hyperexcitability in response to PIC challenge. Briefly, young female C57BL/6 mice were i.p. injected with PIC, and after 24 h, the brains were analyzed by confocal microscopy. CXCL10 staining of neuronal perikarya and a less intense staining of the neuropil was observed in the hippocampus and cortex. CXCL10 staining was also evident in a subpopulation of astrocytes, whereas microglia were CXCL10 negative. CXCR3, the cognate receptor of CXCL10 was present exclusively on neurons, indicating that the CXCL10/CXCR3 axis operates through an autocrine/paracrine neuronal signaling. Blocking cerebral CXCR3 through intracerebroventricular injection of a specific inhibitor, AMG487, abrogated PIC challenge-induced increase in basal synaptic transmission and long-term potentiation (LTP), as well as the reduction of paired-pulse facilitation (PPF), in the hippocampus. The PIC-mediated abolishment of hippocampal long-term depression (LTD) was also restored after administration of AMG487. Moreover, CXCR3 inhibition attenuated seizure hypersensitivity induced by PIC challenge. The efficacy of AMG487 strongly strengthens the notion that CXCL10/CXCR3 axis mediates the induction of cerebral hyperexcitability by PIC challenge.

## Introduction

Peripheral infections are important comorbid factors for the major neuropathological conditions. For example, peripheral infections exacerbate dementia in Alzheimer's disease (AD) (Murray et al., 1993; George et al., 1997; Nee and Lippa, 1999; Holmes et al., 2003; Holmes, 2013), relapses in multiple sclerosis (MS) (Andersen et al., 1993; Edwards et al., 1998; Buljevac et al., 2002; Libbey and Fujinami, 2010) and seizures (Tellez-Zenteno et al., 2005; Scheid and Teich, 2007; Verrotti et al., 2009). It is generally believed that inflammatory agents generated during the initial innate immune response to the invading microbes, i.e., the acute phase response (APR), are relayed to the brain, and by augmenting the ongoing neuropathology exacerbate disease symptoms. However, the underlying cellular/molecular mechanisms have not been defined.

We have developed a preclinical murine model to study mechanisms by which APR exerts its effects on the brain. In this model, APR is induced by intraperitoneal injection of the epitomic viral mimetic, polyinosinic-polycytidylic acid (PIC). We have demonstrated that PIC challenge induces hyperexcitability of neuronal networks as seen from a profound increase in the basal synaptic transmission and long term potentiation (LTP) in hippocampal slices (Hunsberger et al., 2016), as well as from hypersusceptibility to kainic acid (KA)-induced status epilepticus (Kirschman et al., 2011; Michalovicz and Konat, 2014; Hunsberger et al., 2017). Because neuronal hyperexcitability is an invariable feature of the major neuropathologies (Esclapez et al., 1999; Lehmann et al., 2000; Buljevac et al., 2002; Holmes et al., 2003; Caramia et al., 2004; Palasik et al., 2005; Tellez-Zenteno et al., 2005; Scheid and Teich, 2007; Verrotti et al., 2009; Khedr et al., 2011; Penzes et al., 2011; Rossi et al., 2012; Scharfman, 2012; Huynh et al., 2013; Yener and Basar, 2013; Eikermann-Haerter, 2014), it might provide a mechanistic link for the exacerbating effects of peripheral inflammation on disease progression.

At the molecular level, PIC challenge induces a fulminant but transient increase of several inflammatory cytokines (IFNβ, IL-6, IL1β, and TNFα) and chemokines (CXCL10, CCL2, CXCL9, CCL7, and CCL12) in the blood (Michalovicz and Konat, 2014; Petrisko and Konat, 2017). This “cytokine storm” in turn, leads to a robust generation of CXCL10 in the hippocampus, whereas other major inflammatory mediators are either only slightly elevated (CXCL1, CXCL2, CXCL9, IL-6), or unchanged (IL-1β, TNFα) (Petrisko and Konat, 2017). The expression of the *Cxcl10* mRNA is also massively upregulated in the hippocampus following PIC challenge (Michalovicz and Konat, 2014), indicating that CXCL10 is produced *in situ* in the brain.

Because CXCL10 is a potent modulator of neuronal activity (Nelson and Gruol, 2004; Vlkolinsky et al., 2004; Cho et al., 2009), it seems plausible that it might be a putative molecule, which acting through its cognate receptor, CXCR3, drives the development of hyperexcitability. This is congruent with the emerging role of CXCL10 as an important player in diverse neuroinflammatory and neurodegenerative diseases (Michlmayr and McKimmie, 2014).

The present study was undertaken to identify cellular origin of cerebral CXCL10 production instigated by PIC challenge (Petrisko and Konat, 2017). We also appraised the cellular origin of CXCR3. Subsequently, we assessed whether the CXCR3 axis mediates PIC-induced alterations of neuronal activity, i.e., increased basal synaptic transmission and plasticity (Hunsberger et al., 2016), as well as seizure hypersensitivity (Kirschman et al., 2011; Michalovicz and Konat, 2014; Hunsberger et al., 2017).

## Materials and Methods

### Animals

Eight-week old female C57BL/6J mice obtained from Charles River Laboratories (Wilmington, MA) were housed with free access to food and water in a humidity- and temperature-controlled rooms under a 12:12 h light-dark cycle. Female mice were used to be consistent with previous studies (Kirschman et al., 2011; Michalovicz and Konat, 2014; Hunsberger et al., 2016, 2017; Petrisko and Konat, 2017). Mice in all experimental groups were matched by weight prior to treatments. All experimental procedures were approved by the West Virginia University and Auburn University Animal Care and Use Committee and conducted in compliance with the guidelines published in the NIH Guide for the Care and Use of Laboratory Animals.

### Drug Administration

Peripheral APR was induced by a single i.p. injection of 12 mg/kg of ultrapure PIC (Invivogen, San Diego, CA) in 100 μL of saline. Mice injected with equivolume saline served as controls. To verify successful PIC injection, the development of sickness behavior was assessed after 24 h by bodyweight loss (Cunningham et al., 2007).

To block CXCR3, a specific inhibitor, AMG487 (Tocris Bioscience, Minneapolis, MN), was administered by intracerebroventricular (i.c.v.) injection 2 h prior to PIC challenge. Briefly, mice were anesthetized with isoflurane (1.9%-3.4% inhalation; continuous) and immobilized on a stereotaxic frame (Kopf, Tujunga CA). Three mg/kg of AMG-487 (Tocris, Bristol, UK) in 5 μL of artificial cerebrospinal fluid containing 20% of DMSO (ACSF/DMSO) was delivered gradually (0.5 μl/min) into the cerebral ventricles through a 26 s-gauge needle as a bilateral injection of 2.5 uL per side. Mice injected with equivolume ACSF/DMSO served as controls. The coordinates from bregma were: anteroposterior: −0.45 mm, mediolateral: ±0.95 mm, and dorsoventral: −2.6 (Paxinos and Franklin, 2001). The needle was left in place for 5 min to minimize back-flux of the injectate.

Three experimental groups were analyzed: CON group consisting of mice injected with ACSF/DMSO (i.c.v.) and saline (i.p.), PIC group consisting of mice injected with ACSF/DMSO (i.c.v.) and PIC (i.p.) and AMG + PIC group consisting of mice injected with AMG487 (i.c.v.) and PIC (i.p.).

### Microscopy

Twenty-four hours after PIC challenge, mice were deeply anesthetized by i.p. injection of 65 mg/kg of pentobarbital (Beauthanasia, Patterson Veterinary, Devens, MA), sacrificed by pneumothorax, and transaortically perfused with saline followed by 4% paraformaldehyde. The brains were dissected, cryoprotected, and cut into 30 μm coronal sections using the HM450 Sliding Microtome (Thermo Fisher Scientific, Waltham, MA USA). Free-floating immunofluorescent staining of CXCL10, NeuN, GFAP, and Iba1 was performed as previously described (Michalovicz et al., 2015). Briefly, sections were blocked in PBS containing 5% FBS and 0.2% Triton-X 100, and probed with primary antibody followed by secondary antibody. For CXCR3 localization, Triton X-100 concentration was increased to 0.4%. Additionally, for microglia co-staining, anti-CD11b antibody was used to provide compatibility with CXCR3 antibody. Sections were incubated with anti-Cd11b antibody for 96 h at 4°C with rat-anti-CXCR3 being added for the last 12 h. Sections were mounted to slides with Prolong Gold (Thermo Fisher Scientific, Waltham, MA, USA), and imaged using the Nikon A1R Confocal microscope (Nikon Instruments, Melville, NY).

Each cell-type specific stain with CXCL10 or CXCR3 was performed in triplicate. The acquisition settings for CXCL10 and CXCR3 remained the same throughout all experiments. Acquisition settings changed for each cell specific antibody but remained consistent throughout the experiment.

To provide detailed acquisition of cell morphology, as well as CXCL10 and CXCR3 co-expression, Z-stacks were taken with a 60× objective every 0.125 μm. 3D projections were rendered using NIS Elements Advanced Research imaging software (Nikon Instruments, Melville, NY), and the background was subtracted. The images were further analyzed by IMARIS Image Analysis Software (Bitplane Inc., Concord, MA) to reduce false co-staining resultant from the apposition of cells (see Video S1).

Epifluorescent images of cerebral hemispheres were captured using the Olympus VS12 Slide Scanner (Olympus Co, Center Valley, PA) at 10× magnification. Images were imported into NIS Elements, and deconvoluted using the 2D Richardson-Lucy algorithm.

### Antibodies

Primary and secondary antibodies used in this study are shown in Tables 1, 2, respectively. For the co-localization of CXCL10 the primary antibodies were: anti-CXCL10 (AF-466-NA), anti-NeuN (MAB377), anti-GFAP (Z0334), and anti-Iba1 (019-19741). Secondary antibodies were: anti-goat conjugated with Alexa Fluor 555 (A21432), anti-mouse conjugated with Alexa Fluor 488 (A21202) and anti-rabbit conjugated with Alexa Fluor 488 (A21206). For the co-localization of CXCR3 the primary antibodies were: anti-CXCR3 (NBP2-41250), anti-NeuN (MAB377), anti-GFAP (Sc-33673), and anti-CD11b conjugated with BD Horizon BV480 (566117). Secondary antibodies were: anti-rabbit conjugated with Alexa Fluor 555 (A21206), and anti-mouse conjugated with Alexa Fluor 555 (A21202).

**Table 1:** Primary antibodies.

**Antibody**	**Host**	**Clonality**	**Manufacturer**	**Catalog #**	**Dilution**
Anti-CXCL10	Goat	Polyclonal	R&D systems, Minneapolis, MN, USA	AF-466-NA	1:50
Anti-CXCR3	Rabbit	Polyclonal	Novus biologicals, centennial, CO, USA	NBP2-41250^*^	1:2,000
Anti-CXCR3	Rabbit	Polyclonal	Novus Biologicals, centennial, CO, USA	NB100-56404	1:200
Anti-CXCR3	Rabbit	Polyclonal	ABclonal, Woburn, MA, USA	A2939	1:200
Anti-NeuN	Mouse	Monoclonal (clone A60)	EDM Millipore, Burlington, MA, USA	MAB377	1:500
Anti-GFAP	Rabbit	Polyclonal	Dako, Santa Clara, CA, USA	Z0334	1:500
Anti-GFAP	Mouse	Monoclonal (clone 2E1)	Santa Cruz, Santa Cruz, CA, USA	Sc-33673	1:200
Anti-Iba1	Rabbit	Polyclonal	Wako, Richmond, VA, USA	019-19741	1:1,000
Anti-CD11b conjugated to BV480	Rat	Monoclonal (clone M1/70)	BD Biosciences, Franklin Lakes, NJ, USA	566117	1:1,000

* *Peptide affinity purified*.

**Table 2:** Secondary antibodies.

**Antibody**	**Manufacturer**	**Catalog #**	**Dilution**
Donkey anti-Mouse IgG conjugated with Alexa Fluor 488	Thermo Fisher Scientific, Waltham, MA, USA	A21202	1:1,000
Donkey anti-Rabbit IgG conjugated with Alexa Fluor 488	Thermo Fisher Scientific, Waltham, MA, USA	A21206	1:1,000
Donkey anti-Goat IgG conjugated with Alexa Fluor 555	Thermo Fisher Scientific, Waltham, MA, USA	A21432	1:1,000
Donkey anti-Rabbit IgG conjugated with Alexa Fluor 555	Thermo Fisher Scientific, Waltham, MA, USA	A31572	1:1,000

### Extracellular Field Potential Recording

Twenty-four hours after PIC injection, animals were euthanized with carbon dioxide, and 350-μm thick coronal slices through the dorsal hippocampus were prepared using a Leica VT1200S Vibratome (Leica Microsystems, Wetzlar, Germany). For all recordings, a bipolar stimulating electrode was placed in the Schaffer collateral pathway, and a recording pipette was placed in the stratum radiatum of CA1 to record field excitatory post-synaptic potentials (fEPSPs). Electrophysiological measurements, i.e., basal synaptic transmission, paired-pulse facilitation (PPF), long-term potentiation (LTP) and long-term depression (LTD), were performed as previously described (Parameshwaran et al., 2013; Bhattacharya et al., 2015, 2017). Input-output responses were represented by fEPSP slopes and fiber volley amplitudes at increasing stimulus intensities. Basal synaptic transmission was determined by the slope of the linear regression of fEPSP slopes plotted as a function of fiber volley amplitudes. For paired pulse facilitation, paired stimuli were administered at various intervals, and the paired pulse ratio was measured by comparing the slope of the second fEPSP to the slope of the first fEPSP. LTP was induced after at least 10 min of stable baseline using a theta burst stimulation (TBS) protocol consisting of 10 bursts of stimuli, each containing four pulses at 100 Hz, with an interburst interval of 200 ms, and 20 s between the five individual sweeps. LTD was induced using two sweeps of low frequency stimuli (LFS) consisting of 900 pulses at 1 Hz delivered at an interval of 10 min and preceded by at least 10 min of stable baseline. The data were recorded online using the WinLTP software (University of Bristol, UK) (Anderson and Collingridge, 2007).

### Induction of Seizures

Twenty-four hours after PIC injection, seizures were induced by kainic acid (KA) as previously described (Kirschman et al., 2011; Michalovicz and Konat, 2014; Hunsberger et al., 2017). Briefly, mice were subcutaneously (s.c.) injected with 20 mg/kg of KA (Sigma Chemical Co., St. Louis, MO) in saline. Seizure severity was assessed by blinded observers in 5 min intervals for 2 h. The behavioral scores were as follows: 0, no response; 1, immobility; 2, rigid posture; 3, scratching/circling/head bobbing; 4, forelimb clonus/rearing/falling; 5, repetitious pattern; 6, severe tonic-clonic seizures (Morrison et al., 1996). Cumulative seizure scores were calculated as the summation of all scores over the entire period of status epilepticus.

### Statistical Analysis

Comparison of the three experimental groups, i.e., CON, PIC, and AMG + PIC was performed by ANOVA to assess group effect. For measurements involving within-subject measurements, a repeated measures ANOVA (RMANOVA) was performed. Significant omnibus RMANOVA interactions were then analyzed at each within-subject variable using an ANOVA. Only significant omnibus differences at *P* < 0.05 were followed by between group comparisons using Tukey *post-hoc* tests, the results of which are shown in the graphs. All data are expressed as means ± SEM.

## Results

**Figure 1:**
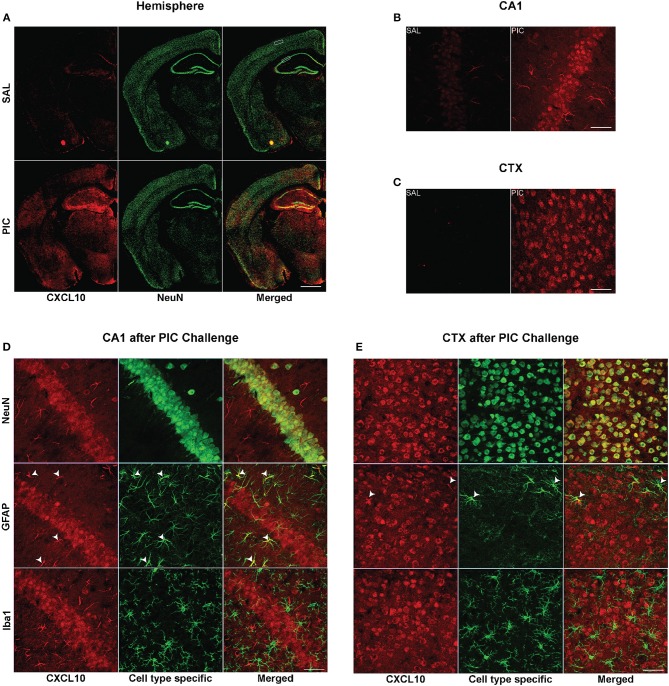
Cellular origin of cerebral CXCL10 generation following PIC challenge. Mice were i.p. injected with 12 mg/kg of PIC or saline (SAL), and after 24 h, analyzed by immunohistochemistry. Upper panels: CXCL10 generation induced by PIC challenge. **(A)** Epiflourescent images of coronal sections of cerebral hemispheres. The upper and lower rectangles delineate positions of regions analyzed in **(B–E)**, i.e., cortical layers 2/3 (CTX) and hippocampal cornu ammonis 1 (CA1), respectively. **(B,C)** Confocal images of CA1 and motor CTX, respectively. CXCL10 was labeled with anti-CXCL10 antibody (red), whereas neurons were labeled with anti-NeuN antibody (green). Lower panels: Cell-specific expression of CXCL10 in the CA1 and CTX of PIC-challenged mice. **(D,E)** Confocal images of the CA1 and CTX, respectively. CXCL10 was labeled with anti-CXCL10 antibody (red). Anti-NeuN (green), anti-GFAP (green) and anti-Iba1 (green) antibodies were used to identify neurons, astrocytes and microglia, respectively. Arrowheads indicate CXCL10^+^ astrocytes. Epifluorescent images **(A)** were captured at 10×, while confocal images **(B–E)** were captured at 60× magnification. Scale bars represent 500 and 50 μm, respectively.

Immunofluorescent analysis performed 24 h after PIC challenge revealed intense CXCL10 staining throughout the brain, whereas a negligible staining was observed in the brain of saline-injected mice (Figure 1A). As shown in Figures 1B,C the same PIC-induced increase in CXCL10 staining was found in discrete brain regions, i.e., hippocampal cornu ammonis 1 (CA1) and motorcortex layers 2/3 (CTX). Subsequent confocal analysis of CA1 and CTX revealed that most of CXCL10 staining was localized to neuronal perikarya (Figures 1D,E). The staining extended into the proximal parts of neuronal processes. However, the intensity of CXCL10 staining among NeuN-positive neurons varied. This variation was particularly well-demonstrable in the cortex (Figure 1E) where individual neurons were more discernible. Also, a subpopulation of astrocytes expressed CXCL10. The staining was evident in the cell bodies and cytoplasmic processes. In contrast, no CXCL10 staining co-localized with Iba1-positive microglia. Although discrete regions of microglia occasionally featured apparent co-staining, the analysis of multiple sections in 3D renderings using Nikon and IMARIS software revealed this co-staining to be false-positive due to close apposition of microglia to CXCL10^+^ neurons and/or astrocytes (Video S1). The same cell-specific co-localization of CXCL10 was observed at shorter time intervals after PIC challenge, i.e., 6 and 12 h (not shown).

**Figure 2:**
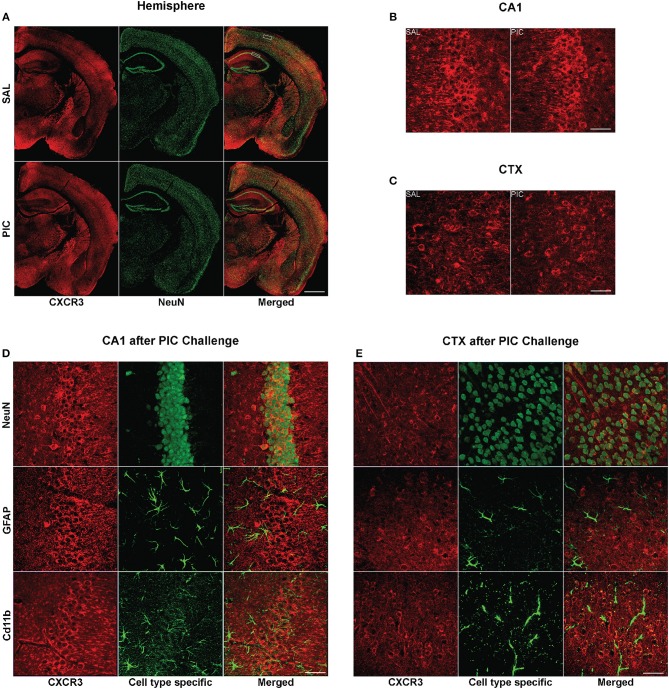
Cellular expression of cerebral CXCR3. Mice were i.p. injected with 12 mg/kg of PIC or saline (SAL), and after 24 h, analyzed by immunohistochemistry. Upper panels: CXCR3 expression following PIC challenge. **(A)** Epiflourescent images of coronal sections of cerebral hemispheres. The upper and lower rectangles delineate the positions of regions analyzed in **(B–E)**, i.e., cortical layers 2/3 (CTX) and hippocampal cornu ammonis 1 (CA1), respectively. **(B,C)** Confocal images of CA1 and motor CTX, respectively. CXCR3 was labeled with anti-CXCR3 antibody (red), whereas neurons were labeled with anti-NeuN (green). Lower panels: Cell-specific expression of CXCR3 in the CA1 and CTX of PIC-challenged mice. **(D,E)** Confocal images of the CA1 and CTX, respectively. CXCR3 was labeled with anti-CXCR3 antibody (red). Anti-NeuN (green), anti-GFAP (green) and anti-CD11b (green) antibodies were used to identify neurons, astrocytes and microglia, respectively. Epifluorescent images **(A)** were captured at 10×, while confocal images **(B–E)** were captured at 60× magnification. Scale bars represent 500 and 50 μm, respectively.

As depicted in Figure 2, neurons also expressed the cognate receptor of CXCL10, CXCR3. This neuron specific expression of the receptor was verified using three unrelated antibodies (Figure S1). In congruence with the previously observed lack of upregulation of the *Cxcr3* mRNA expression by PIC challenge (Fil et al., 2011), there was no detectable difference in CXCR3 staining intensity between brain tissue from PIC-injected and saline injected mice (Figures 2A–C). The expression was confined to the cell surface of neuronal perikarya. Although, CXCR3 staining was also evident in neuronal processes, particularly, in the cortex. CXCR3 expression was evident throughout the brain. No apparent CXCR3 staining was detectable in either astrocytes or microglia.

**Figure 3:**
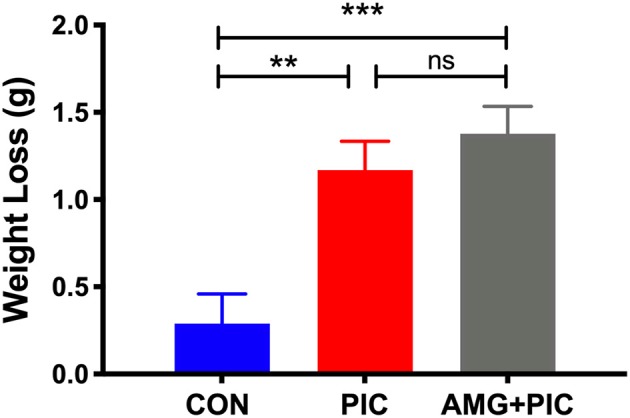
Effect of CXCR3 inhibition on weight loss in PIC-challenged mice. Mice were i.c.v. injected with AMG487 (3 mg/kg) in ACSF/DMSO or with ACSF/DMSO alone. Two hours later, mice were weighed, and i.p. injected with 12 mg/kg of PIC in saline, or saline alone. Twenty-four hours after PIC injection, mice were weighed again, and the amount of weight lost was compared [ANOVA: *F*_(2,70)_ = 10.4, *p* < 0.0001]. The following groups were analyzed: CON; injected with ACSF/DMSO and saline, PIC; injected with ACSF/DMSO and PIC, and AMG+PIC; injected with AMG487 and PIC. Bars represent means ± SEM from 5 to 6 mice per group. **p ≤ 0.01, ***p ≤ 0.001; ns, not significantly different.

To assess the role of the CXCL10/CXCR3 axis in the induction of neuronal hyperexcitability, we used i.c.v. injection of a specific inhibitor, AMG487, to block CXCR3 signaling before challenging the animals with PIC. To determine whether the inhibitor alters the PIC-induced sickness behavior, we measured the loss of body weight 24 h after PIC challenge. As previously observed (Cunningham et al., 2007), the injection of saline and PIC induced ~1.5 and 5.9% body loss as assessed 24 h later (Figure 3). No additional increase in the weight loss was found in mice injected with PIC and AMG487.

**Figure 4:**
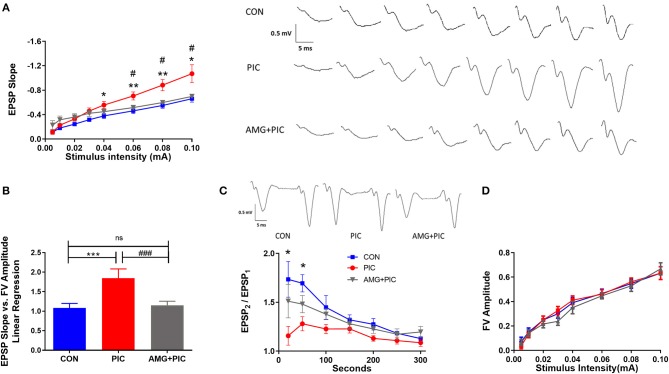
Effect of CXCR3 inhibition on synaptic transmission in hippocampal slices. Mice were i.c.v. injected with AMG487 (3 mg/kg) in ACSF/DMSO or with ACSF/DMSO alone. Two hours later, mice received an i.p. injection of 12 mg/kg of PIC in saline, or saline alone. Twenty-four hours after PIC injection, basal synaptic transmission was assessed in hippocampal slices. The following groups were analyzed: CON; injected with ACSF/DMSO and saline, PIC; injected with ACSF/DMSO and PIC, and AMG + PIC; injected with AMG487 and PIC. **(A)** Basal synaptic transmission represented by the fEPSP slope measured at increasing stimulus intensities [RMANOVA, Group*Intensity: *F*_(14,105)_ = 5.5, *p* < 0.0001]. **(B)** Basal synaptic transmission represented by the slope of the linear regression between fEPSP slope (Y axis) and FV amplitude (X axis) [ANOVA: *F*_(2,15)_ = 6.5, *p* = 0.009]. **(C)** Paired-pulse facilitation expressed as the change of ratio of the second stimulus fEPSP to the first stimulus fEPSP slope plotted as a function of interstimulus interval [RMANOVA, Group*Seconds: *F*_(12,72)_ = 2.9, *p* = 0.0026]. Representative traces at the 20 ms interpulse interval are shown. **(D)** Fiber volley (FV) analysis represented by the FV amplitude measured at increasing stimulus intensities [RMANOVA, Group*Intensity: *F*_(14,105)_ = 0.71, *p* = 0.76]. Symbols represent means ± SEM from 5 to 6 mice per group. *Represents significant difference between CON and PIC; ^#^represents significant difference between PIC and AMG + PIC; *^/#^*p* ≤ 0.05, **^/##^*p* ≤ 0.01, ***^/###^*p* ≤ 0.001; ns, not significantly different.

Basal synaptic transmission was evaluated in hippocampal slices 24 h after PIC challenge (Figure 4). At stimulus intensities from 40 to 100 μA, basal synaptic transmission increased by ~42% in the slices from PIC-challenged compared to control mice. CXCR3 blockade abrogated this increase (Figure 4A). To determine whether there is a relationship between pre- and post-synaptic responses, fiber volley, which represents the presynaptic action potential in response to stimulus, was compared to post-synaptic responses represented as field excitatory post-synaptic potentials (fEPSPs) using linear regression analysis. PIC challenged mice exhibited increased post-synaptic responses to the same fiber volley amplitude compared to control mice, whereas blockade of CXCR3 mitigated this increase (Figure 4B).

To determine if the hyperexcitability of the neurons following PIC challenge are due to presynaptic modifications, the probability of neurotransmitter release was examined by measuring paired pulse facilitation (PPF). There was a significant reduction in PPF at the short stimulus intervals in slices from PIC-challenged vs. control mice (Figure 4C), indicating an increase in presynaptic release probability. The drop in EPSP_2_/EPSP_1_ was 35 and 25% at 25 and 50 ms stimulus intervals, respectively. No significant changes were evident at longer stimulus intervals. The PIC challenge-induced decrease in PPF was attenuated by the pretreatment of mice with AMG487. To test if the changes in PPF in PIC-challenged mice are due to presynaptic axonal recruitment, fiber volley amplitude vs. stimulus intensities was compared. PIC challenge had no effect on the fiber volley amplitude across various stimulus intensities (Figure 4D), which indicates no changes in presynaptic axon recruitment.

**Figure 5:**
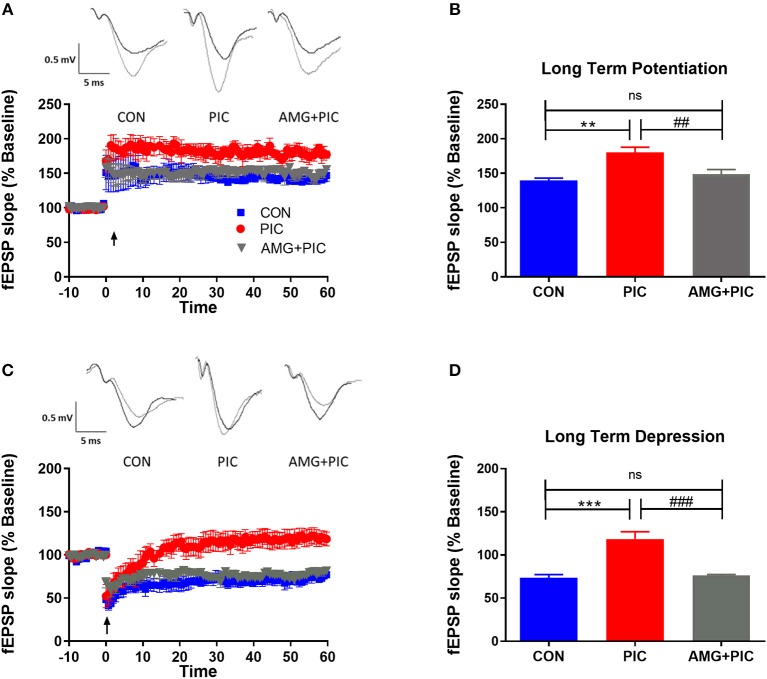
Effect of CXCR3 inhibition on synaptic plasticity in hippocampal slices. Mice were i.c.v. injected with AMG487 (3 mg/kg) in ACSF/DMSO or ACSF/DMSO. Two hours later, mice were i.p. injected with 12 mg/kg of PIC in saline or saline. Twenty-four hours after PIC injection, synaptic plasticity was assessed in hippocampal slices. The following groups were analyzed: CON; injected with ACSF/DMSO and saline, PIC; injected with ACSF/DMSO and PIC, and AMG+PIC; injected with AMG487 and PIC. **(A)** Long term potentiation (LTP) represented by percent change in fEPSP slope over time. **(B)** LTP represented by fEPSP slope during 50–60 min following LTP induction [ANOVA: *F*_(2,12)_ = 12.4, *p* = 0.0012]. **(C)** Long term depression (LTD) represented by percent change in fEPSP slope over time. **(D)** LTD represented by fEPSP slope during 50–60 min following LTD induction [ANOVA: *F*_(2,12)_ = 21.7, *p* < 0.0001]. Symbols represent means ± SEM from 5 to 6 mice per group. *Represents significant difference between CON and PIC; ^#^represents significant difference between PIC and AMG + PIC; *^/#^*p* ≤ 0.05, **^/##^*p* ≤ 0.01, ***^/###^*p* ≤ 0.001; ns, not significantly different.

To determine if these alterations in basal synaptic transmission could result in altered synaptic plasticity, LTP and LTD were assessed. PIC challenge significantly enhanced LTP (Figures 5A,B). When expressed as average fEPSP slope during 50–60 min following LTP induction, the enhancement amounted to 32% over the control value. AMG487 pretreatment negated this enhancement. PIC-challenged mice failed to exhibit LTD (Figures 5C,D), whereas CXCR3 inhibition with AMG487 restored LTD to levels similar to control mice.

**Figure 6:**
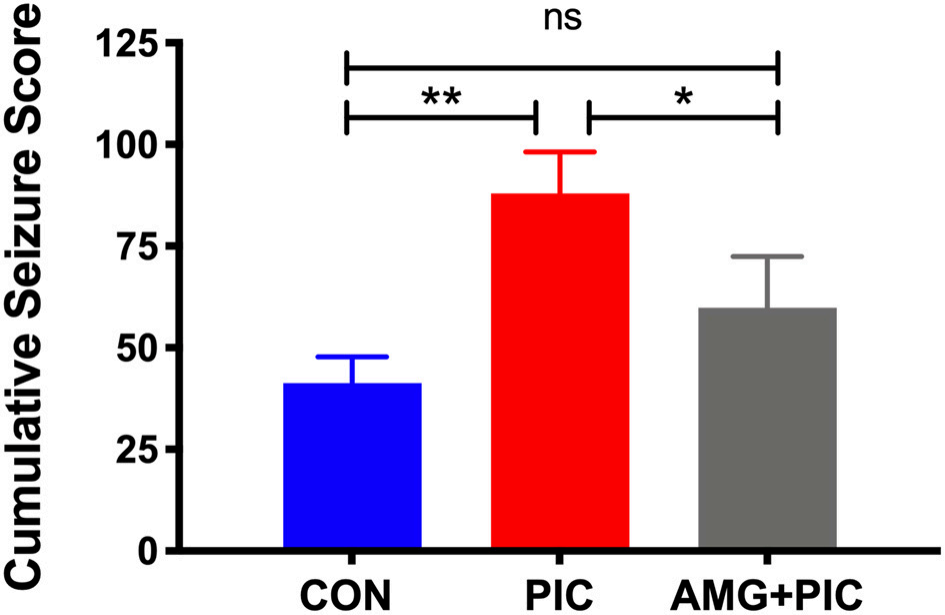
Effect of CXCR3 inhibition on seizure hypersensitivity induced by PIC challenge. Mice received an i.c.v. injection of AMG 487 (3 mg/kg) in ACSF/DMSO or ACSF/DMSO alone. Two hours later, mice were i.p. injected with 12 mg/kg of PIC in saline, or saline alone. Twenty-four hours after PIC injection, status epilepticus (SE) was induced by subcutaneous (s.c.) injection of 12 mg/kg of kainic acid (KA). The following groups were analyzed: CON; injected with ACSF/DMSO and saline, PIC; injected with ACSF/DMSO and PIC, and AMG + PIC; injected with AMG487 and PIC. Seizures were expressed as cumulative seizure scores [ANOVA: *F*_(2,9)_ = 13.86, *p* = 0.0018]. Symbols represent means ± SEM from 3 to 6 mice per group. *p ≤ 0.05, **p ≤ 0.01; ns, not significantly different.

To further verify involvement of the CXCL10/CXCR3 axis in PIC-induced neuronal hyperexcitability, we used an *in vivo* model of temporal lobe seizures utilizing an excitatory neurotoxin, kainic acid (KA). Hippocampal neurons are the primary targets of KA, and therefore, the hippocampus is the ictal site of KA-induced seizures (Ben-Ari, 1985; Ben-Ari and Cossart, 2000; Vincent and Mulle, 2009). As previously demonstrated (Kirschman et al., 2011; Michalovicz and Konat, 2014; Hunsberger et al., 2017), PIC challenge increased the susceptibility of mice to KA-induced seizures (Figure 6). PIC-challenged mice featured a 47% increase in cumulative seizure score vs. control mice. CXCR3 blockade with AMG-487 attenuated this PIC-mediated increase.

## Discussion

We have previously shown that PIC challenge robustly upregulates cerebral expression of the *Cxcl10* gene at the message (Fil et al., 2011; Michalovicz and Konat, 2014) and protein (Petrisko and Konat, 2017) levels. Here, we found neurons to be the predominant cell type responsible for CXCL10 generation in the brain. Interestingly, neuronal CXCL10 production has been shown to be induced by viral encephalitides (Rappert et al., 2004; Klein et al., 2005; Chai et al., 2015), indicating a commonality of neuronal response to systemic and central viral challenge. In concordance with previous study (Xia et al., 2000), only a subpopulation of astrocytes expressed CXCL10, although the contribution of these cells to the global production of the chemokine seems to be negligible. Resting microglia do not express CXCL10, but the expression is induced in microglia activated by viral infection of the brain (Chai et al., 2015). PIC challenge did not elicit microglial expression of CXCL10, indicating no development of proinflammatory phenotype of microglia. Our previous studies showing no effect of PIC challenge on the expression of cerebral iNOS (Konat et al., 2009) and CX3CL1 (Fil et al., 2011), the indices of neuroinflammation, buttress this contention.

CXCR3 is expressed constitutively on neurons and neuronal processes in the brain (Xia et al., 2000; Wang et al., 2017; Piotrowska et al., 2018). Here, we found a similar pattern of expression of the receptor. Neuronal expression of both the receptor and its ligand indicates that the CXCL10/CXCR3 axis operates through an autocrine/paracrine neuronal signaling. Moreover, we found CXCR3 to be restricted to neurons, as both astrocytes and microglia were CXCR3-negative. Previously, CXCR3 expression was found in reactive, but nor quiescent astrocytes and microglia in a variety of CNS pathologies (Tanuma et al., 2006; Goldberg et al., 2010). Thus, the lack of glial CXCR3 expression provides further support for the notion that PIC challenge does not promote proinflammatory transformation of astrocytes and microglia.

In concordance with our previous electrophysiological study (Hunsberger et al., 2016), PIC challenge increased basal synaptic transmission, resulting in hyperexcitability of hippocampal circuits. The increased basal synaptic transmission likely occurred due to both increased presynaptic release and enhanced post-synaptic activity. The increase in presynaptic release in PIC-challenged mice possibly resulted from altered release mechanisms at the synapse, because the data suggests no changes in presynaptic axon recruitment. Future studies will assess whether the enhanced post-synaptic activity is due to increased recruitment of glutamate receptors at the synapse or increased ion channel activity, or possibly both. Of note, we showed that alterations in both basal synaptic transmission and synaptic plasticity, as well as PIC-induced seizure hypersensitivity, are abolished by the inhibition of CXCR3. These novel findings strongly indicate that the CXCL10/CXCR3 axis governs the induction of neuronal hyperexcitability. These results dovetail with previous *in vitro* studies that found CXCL10 to increase electrical activity of neurons in culture (Nelson and Gruol, 2004; Cho et al., 2009). Several possible mechanisms can be envisaged. For example, increased neuronal activity might result from the suppression of inhibitory GABAergic transmission, as a consequence of the downregulation of GAD65/67 and two GABAergic receptors, GABA_B_R1 and GABA_A_Rα2 (Cho et al., 2009). Interestingly, we found PIC challenge to downregulate the GABA receptor subunit ε (GABRE) (Michalovicz and Konat, 2014). Moreover, the ligation of neuronal CXCR3 activates the ERK1/2 pathway (Xia et al., 2000), which phosphorylates gephyrin leading to the disruption of GABAergic synapses and their decreased transmission (Tyagarajan et al., 2013). Increased neuronal activity might also be attributed to the enhancement of excitatory glutamatergic transmission, as CXCL10 upregulates the expression of two glutamatergic receptors, NMDAR1 and mGluR2/3 (Cho et al., 2009). It is likely that both the attenuation of GABAergic and the enhancement of excitatory transmission contribute to shifting the neuronal balance toward excitation. Furthermore, additional, as yet unexplored, mechanisms might also mediate the development of hyperexcitability triggered by the neuronal CXCL10/CXCR3 axis signaling. These mechanisms will be explored in future studies.

## Conclusion

In conclusion, we have demonstrated that anti-viral APR activates neuronal CXCL10/CXCR3 axis in the brain, and that this activation elicits hyperexcitability of neuronal circuits. Our results strongly suggest that the CXCL10/CXCR3 axis may play a critical role in the comorbid effect of peripheral viral infections on the progression of major neuropathological diseases.

## Data Availability Statement

The datasets generated for this study are available on request to the corresponding author.

## Ethics Statement

The animal study was reviewed and approved by West Virginia University Care and Use Committee, Morgantown, WV, USA; and Auburn University Animal Care and Use Committee, Auburn, AL, USA.

## Author Contributions

MR and GK conceived and designed the experiments and wrote the manuscript. VS aided in experimental design. TP, JB, SES, SS, PP, YD, HH, and RH performed the experiments, analyzed the data, or edited the manuscript.

### Conflict of Interest

The authors declare that the research was conducted in the absence of any commercial or financial relationships that could be construed as a potential conflict of interest.

## Supplementary Material

Figure S1 Verification of neuronal expression of CXCR3. Cortical sections form control mice were processed as described in Materials and Methods, and probed with three unrelated anti-CXCR3 antibodies, i.e., NBP2-41250, NB100-56404, and A2939 (Table 1). Neuronal bodies were stained with anti-NeuN antibody. Following secondary antibody staining (Materials and Methods), confocal images were captured at 60× magnification. Scale bar represents 50 μm.

Video S1 Resolution of false co-localization of CXCL10 in microglia. Mice were i.p. injected with PIC, and coronal sections through the CA1 hippocampal region were analyzed after 24 h. The sections were double stained with anti-CXCL10 (red) and anti-Iba1 antibodies (green), and confocal images were analyzed by IMARIS as described in Materials and Methods. A few segments of microglial cells seemed to co-express CXCL10 (yellow). However, rotation of the image revealed that microglial CXCL10 staining resulted from under- or overlapping CXCL10^+^ neurons and astrocytes.
