# Supplementary figures and images for: Neuronal CXCL10/CXCR3 Axis Mediates the Induction of Cerebral Hyperexcitability by Peripheral Viral Challenge
Source: Front Neurosci. 2020 Mar 24;14:220. doi: 10.3389/fnins.2020.00220 (PMC7105801; doi:10.3389/fnins.2020.00220)

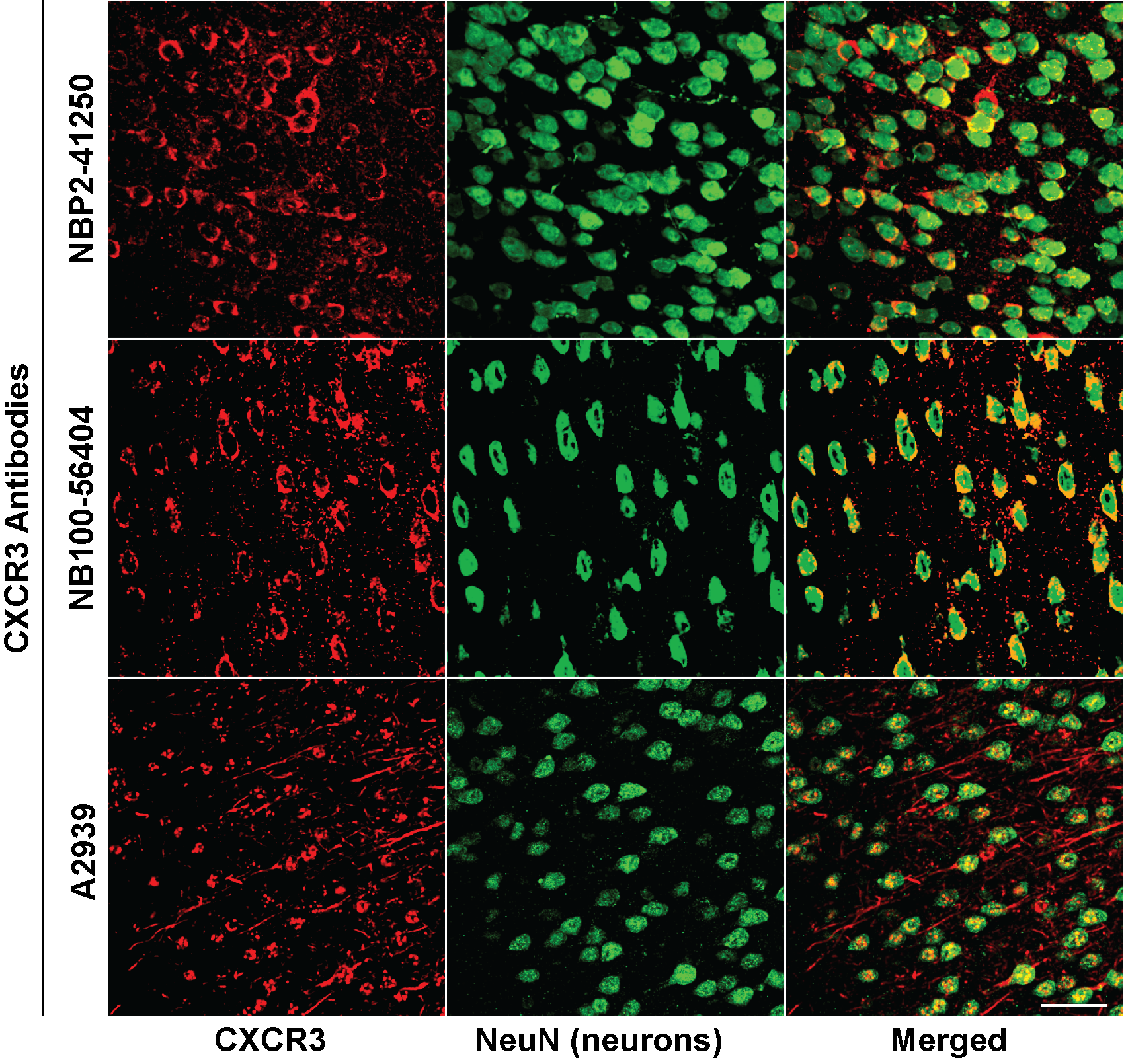

Supplement: Figure S1 — Verification of neuronal expression of CXCR3. Cortical sections form control mice were processed as described in Materials and Methods, and probed with three unrelated anti-CXCR3 antibodies, i.e., NBP2-41250, NB100-56404, and A2939 (Table 1). Neuronal bodies were stained with anti-NeuN antibody. Following secondary antibody staining (Materials and Methods), confocal images were captured at 60× magnification. Scale bar represents 50 μm. [file Image_1.TIFF]
